# Adoption of Artificial Intelligence–Enabled Robots in Long-Term Care Homes by Health Care Providers: Scoping Review

**DOI:** 10.2196/55257

**Published:** 2024-08-27

**Authors:** Karen Lok Yi Wong, Lillian Hung, Joey Wong, Juyoung Park, Hadil Alfares, Yong Zhao, Abdolhossein Mousavinejad, Albin Soni, Hui Zhao

**Affiliations:** 1 IDEA Lab University of British Columbia Vancouver, BC Canada; 2 College of Nursing University of Arizona Tucson, AZ United States; 3 School of Nursing James Madison University Harrisonburg, VA United States

**Keywords:** artificial intelligence, robot, long-term care home, health care provider, scoping review, person-centered care

## Abstract

**Background:**

Long-term care (LTC) homes face the challenges of increasing care needs of residents and a shortage of health care providers. Literature suggests that artificial intelligence (AI)–enabled robots may solve such challenges and support person-centered care. There is a dearth of literature exploring the perspectives of health care providers, which are crucial to implementing AI-enabled robots.

**Objective:**

This scoping review aims to explore this scant body of literature to answer two questions: (1) what barriers do health care providers perceive in adopting AI-enabled robots in LTC homes? (2) What strategies can be taken to overcome these barriers to the adoption of AI-enabled robots in LTC homes?

**Methods:**

We are a team consisting of 3 researchers, 2 health care providers, 2 research trainees, and 1 older adult partner with diverse disciplines in nursing, social work, engineering, and medicine. Referring to the Joanna Briggs Institute methodology, our team searched databases (CINAHL, MEDLINE, PsycINFO, Web of Science, ProQuest, and Google Scholar) for peer-reviewed and gray literature, screened the literature, and extracted the data. We analyzed the data as a team. We compared our findings with the Person-Centered Practice Framework and Consolidated Framework for Implementation Research to further our understanding of the findings.

**Results:**

This review includes 33 articles that met the inclusion criteria. We identified three barriers to AI-enabled robot adoption: (1) perceived technical complexity and limitation; (2) negative impact, doubted usefulness, and ethical concerns; and (3) resource limitations. Strategies to mitigate these barriers were also explored: (1) accommodate the various needs of residents and health care providers, (2) increase the understanding of the benefits of using robots, (3) review and overcome the safety issues, and (4) boost interest in the use of robots and provide training.

**Conclusions:**

Previous literature suggested using AI-enabled robots to resolve the challenges of increasing care needs and staff shortages in LTC. Yet, our findings show that health care providers might not use robots because of different considerations. The implication is that the voices of health care providers need to be included in using robots.

**International Registered Report Identifier (IRRID):**

RR2-doi:10.1136/bmjopen-2023-075278

## Introduction

### Background

Long-term care (LTC) provides various services designed to meet the chronic health and personal care needs of those who can no longer perform daily activities independently [1]. LTC health care providers face challenges to meet the increased demand from older adults and their family caregivers due to a dramatically increasing aging population and growing chronic disease burden [2]. Health care providers in LTC homes often engage in repetitive tasks, many involving physical labor, which could lead to a high risk of job stress, physical or emotional exhaustion, burnout, and high turnover, all of which contribute to a lower quality of care [3-5]. Thus, innovative solutions are required to meet LTC home residents’ health care needs and reduce the workload for health care providers.

Artificial intelligence (AI)–enabled robots have been perceived as a solution to the crisis in LTC homes, where significant labor shortages will accompany rapidly increasing care demand [6-8]. AI-enabled robots have been used to support person-centered care for older adults and attend to the emotional, social, and physical needs of older adults. For example, PARO, a socially assistive robot, can interact with and provide emotional support for patients with dementia [9]. Physically assistive robots can perform tasks such as dressing and sit-to-stand support [10]. Evidence has suggested that using AI-enabled robots in LTC homes could optimize resources, enhance resident outcomes, create patient-centered care, satisfy residents’ needs, and improve health care providers’ workflow [2]. While AI-enabled robots may potentially alleviate the burden on health care workers and enhance efficiency in LTC homes, they also pose risks. Issues associated with AI-enabled robot use were explored in the literature. In LTC homes, the adoption and use of robotics are associated with ethical issues and technological risks such as safety, privacy and data security, liability, and effects on the incumbent workforce [11]. Accordingly, research has been focusing on examining attitudes and perceptions of AI-enabled robots [12].

Recent AI-enabled robot studies evaluate the acceptance of this technology in older users, including in the settings of care facilities, as well as private homes and living lab contexts [13-18]. Findings of the literature show that older adults are generally open to robot assistants, while robots provide social interactions, cognitive stimulation, home-based tasks, personal care, and information management [19,20]. However, very few studies have been focused on measuring the health care providers’ perception of AI use, although their acceptance of AI-enabled robots is crucial to future research and development and the implementation of AI-enabled robots in LTC homes [21]. Some studies have shown that approximately 40% of technologies, such as home health care robots and information systems, have been abandoned in the last 2 decades [22,23]. Several barriers to health care providers’ adoption of AI-enabled robots were explored, including clinicians’ inadequate knowledge [24] and lack of understanding of the sociotechnical aspects of the technology [24]. These barriers lead to a fear of job loss among health care staff, who are concerned about being replaced by robots for repetitive or manual tasks, even when robots are intended to assist rather than replace workers [21,24]. Therefore, understanding the perspectives of health care providers on AI-enabled robot use is crucial, as they can offer the most pertinent insights into the risks and impacts, as well as to understand users’ needs and expectations [25].

This scoping review aims to synthesize and analyze the existing literature on the potential barriers and the strategies to overcome these barriers by adopting AI-enabled robots in LTC homes from the perspectives of health care providers. Two research questions guided the review: (1) what barriers do health care providers perceive in adopting AI-enabled robots in LTC homes? (2) What strategies can be taken to overcome these barriers to the adoption of AI-enabled robots in LTC homes?

To our knowledge, no scoping review has been conducted on this topic. Existing scoping reviews focus on using AI in older adult care or health care, such as promoting shared health care decision-making [26], monitoring diabetes-related parameters [27], and facilitating digital health care interventions [28]. However, these settings are not LTC homes. There are also scoping reviews on LTC homes, such as making decisions about moving into LTC homes [29] and physical rehabilitation in LTC homes [30]. Yet, they are not related to the use of technology. There are scoping reviews about technologies in LTC homes, such as using eHealth to support assessment and decision-making with residents living with dementia in LTC homes [31] and defining the concepts of smart nursing homes and technology-assisted LTC homes [32]. Nevertheless, these reviews are not specifically about AI. Lukkien et al [33] conducted a scoping review about responsible AI, that is, using AI ethically in LTC homes. Yet, the review is from the perspectives of researchers, not health care providers.

The paper addresses the critical gap concerning LTC health care providers’ perspectives in adopting AI robots. Staff perspectives are essential as they directly impact the acceptance, use, and effectiveness of AI technologies in care settings. Our findings highlight the importance of an inclusive approach to engaging LTC staff in robot development and implementation. The practical insights and strategies can empower staff to support the integration of AI technologies into LTC.

To begin with, we will define some terms used in this paper. We have published a protocol for this scoping review and will refer to the definitions of *robot*, *AI*, and *AI-enabled robot* as outlined in the protocol [34]: “*Robots* are mechanical devices that can be of various physical forms and are designed to perform a wide range of tasks.... *AI* is known as ‘the science of making [a] machine or computer to act intelligently’.... The *AI-enabled robot*, or intelligence robot, can be defined as ‘a physically situated intelligent agent in the “real world,” regardless of shape, that can sense and act on its operational environment.’ AI allows robots to (a) present the world symbolically in a way that can be easily understood by computers, (b) understand natural language and explore clear communication required for comfortable social interaction between humans and robots, (c) learn by self-iterative trials and apply that learning to a range of functions, (d) plan and solve problems, (e) generate an answer without complete information, (f) use search algorithms to generate solutions in navigation or search for optimal knowledge representation and (g) improve robotic actions with vision systems in the robots” [35]. *Health care providers* refer to paid staff caring for LTC home residents (eg, nurses, care aides, and allied health professionals). *LTC* refers to “care settings that provide 24-hour personal care support for people with complex needs who are unable to remain at home” [34].

### Theoretical Frameworks

In this scoping review, the Person-Centered Practice Framework (PCPF) [36] and Consolidated Framework for Implementation Research (CFIR) [37] are the supplementary theoretical frameworks that guided our synthesis and analysis of results to explore the barriers and the strategies to overcome these barriers to the adoption of AI-enabled robots in LTC homes.

#### PCPF Philosophy

McCormack and McCance [38] coined *PCPF*. Person-centered care is a care philosophy with people living with dementia [39]. Despite cognitive impairment, person-centered care recognizes that a person living with dementia still has personhood; should be seen as a person; and has diverse needs, such as psychosocial needs, which need to be met by care to achieve the person’s holistic well-being. PCPF provides a framework for understanding the factors influencing the practice of person-centered care with people living with dementia [38,40]. Our review focuses on adopting AI-enabled robots to care for people with dementia. PCPF is, therefore, a good fit for our review. PCPF initially focuses on nursing practice with people living with dementia [38]. However, gradually, it has been adopted by other disciplines, such as social work [41] and rehabilitation [39]. PCPF is an evolving framework since its first publication in 2001; its author continues to enhance it over the years by absorbing lessons from new research and practice [36,38,40]. We have adopted the latest version of PCPF as published by the authors in 2023 [36].

**Figure 1:**
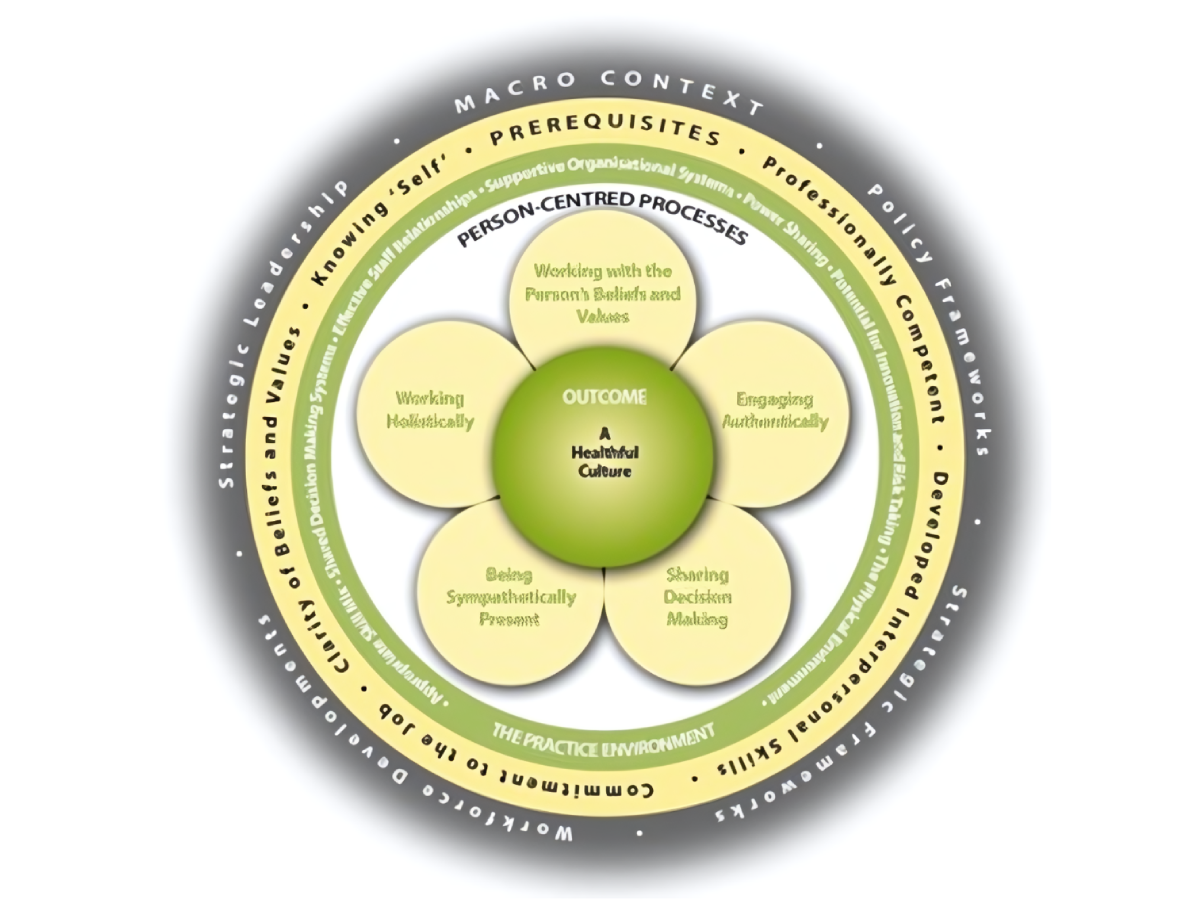
Developing healthful cultures through the development of person-centered practice. Reproduced from McCance and McCormack [36] with permission from Elsevier.

PCPF comprises 5 domains: prerequisite, practice environment, person-centered process, outcome, and macrocontext. Figure 1 presents the framework from the authors’ recent publication in 2023 [36].

PCPF considers that person-centered care practice is shaped not only by factors at the level of the patient and the health care provider but also by factors at the level of organization and society. These domains are interrelated with each other, as elaborated by the authors [36]: “to reach the centre of the framework, one must first take account of the *macrocontext*, followed by consideration of the attributes of staff, as a *prerequisite* to managing the practice *environment*, and in order to engage effectively through the *person-centred processes*. This ordering ultimately leads to the achievement of the *outcome*.” Under each of the 5 domains, there are constructs. There is no hierarchy among the constructs; constructs in the same domain can be related or even overlap [36].

The *prerequisite* domain examines the “attributes” of health care providers required to provide person-centered care, and the constructs under this domain are “being professionally competent, having developed interpersonal skills, being committed to the job, being able to demonstrate clarity of beliefs and values, and knowing self” [36]. The practice *environment* domain looks into “the context,” that is, the care setting and organization, where person-centered care is provided, and under this domain, the constructs include “appropriate skill mix; systems that facilitate shared decision making; the sharing of power; effective staff relationships; organisational systems that are supportive; potential for innovation and risk taking; and the physical environment” [36]. The *person-centered process* domain explores health care providers’ “ways of engaging that are necessary to create connections between persons (living with dementia),” and the constructs under this domain include “working with the person’s beliefs and values; engaging authentically; being sympathetically present; sharing decision making; and working holistically” [36]. The *outcome domain* looks into the “result of effective person-centred practice,” and the authors suggest that effective person-centered practice should “enable human flourishing for those who give care and for those who receive care” [36]. Under this domain, the constructs are that “decision-making is shared, relationships are collaborative, leadership is transformational, and innovative practices are supported” [36]. The *macrocontext* domain refers to regional, national, and international “factors that are strategic and political in nature that influence the development of person-centred cultures” [36]. The constructs under this domain include “policy frameworks, strategic frameworks, workforce developments, and strategic leadership” [36].

#### CFIR Framework

Damschroder et al [42] developed the CFIR by consolidating constructs from 19 implementation frameworks or theories in 2009. The authors updated it by incorporating new literature and feedback in 2022 [43,44]. Our review will refer to the latest version of CFIR, which is freely accessible on the CFIR website [45]. CFIR is a framework for understanding the contextual factors that influence implementation in a clinical setting or organization [44]. Our review concerns the implementation of AI-enabled robots in LTC. Thus, CFIR and our review are a good match. In addition, previous literature suggested that CFIR can be used with other frameworks [44]. In our review, we are using it with PCPF.

According to the authors, one way to use CFIR is to better understand the findings on implementation [44]. We will use CFIR to understand the findings from the literature on health care providers’ perspectives on the barriers to implementing AI-enabled robots and the strategies for overcoming them. The authors suggested 2 approaches to using CFIR to understand the findings on implementation: deductive and inductive. Our study will use a mixed approach, which will be further elaborated.

CFIR comprises 5 domains: innovation, inner setting, outer setting, individuals, and implementation process; there are constructs under each domain, and CFIR has 48 constructs in total [37]. The *innovation domain* explores “the ‘thing’ being implemented,” such as research, programs, policies, and innovations [37]. The constructs under this domain include “innovation source, innovation evidence-base, innovation relative advantage, innovation adaptability, innovation trialability, innovation complexity, innovation design, and innovation cost” [37]. The *outer setting domain* examines “the setting in which the inner setting exists,” such as the health and community organizations [37]. Under this domain, the constructs are “critical incidents, local attitudes, local conditions, partnerships and connections, policies and laws, financing, and external pressure” [37]. The *inner setting domain* looks into “the setting in which the innovation is implemented,” such as an LTC home or a hospital [37]. The constructs under this domain are “structural characteristics, relational connections, communications, culture, tension for change, compatibility, relative priority, incentive systems, mission alignment, available resources, and access to knowledge and information” [37]. The *individuals domain* explores “the roles and characteristics of individuals” involved in implementation [37]. Under this domain, the constructs include “high-level leaders, midlevel leaders, opinion leaders, implementation facilitators, implementation leads, implementation team members, other implementation support, innovation deliverers, and innovation recipients” [37]. The *implementation process domain* examines “the activities and strategies used to implement the innovation” [37]. The constructs under this domain are “teaming, assessing needs, assessing context, planning, tailoring strategies, engaging, doing, reflecting and evaluating, and adapting” [37].

## Methods

### Overview

Scoping reviews are pivotal for identifying and summarizing evidence in emergent fields and highlighting significant themes, contexts, and research gaps [46]. Given the nascent stage of AI-enabled robots in LTC homes, a scoping review was apt for our study. Our interdisciplinary review team was comprised 3 researchers, 2 health care providers, 2 research trainees, and 1 older adult partner. We each brought different expertise: researchers and research trainees brought research knowledge and skills, health care providers brought frontline experiences, and the older adult partner brought in lived expertise. Our group represented diverse backgrounds: nursing, social work, engineering, and medicine. Diverse expertise and backgrounds enriched our team discussions, especially during data analysis. We followed the guidelines on scoping reviews outlined by the Joanna Briggs Institute [47]. We published the objectives, inclusion criteria, and methods of this scoping review in a protocol [34]. We conducted the scoping review over 6 months. As data were synthesized solely from existing literature, ethics approval was not required for this scoping review.

### Search Strategy

We followed the three-step search approach recommended by Joanna Briggs Institute: (1) conducting a preliminary search using 2 databases (MEDLINE and CINAHL) to identify keywords and index terms; (2) using keywords and index terms from the previous step to search selected databases (CINAHL, MEDLINE, PsycINFO, Web of Science, ProQuest, and Google Scholar); and (3) hand-searching the reference lists of selected items.

The participants, context, and concept of our scoping review were as follows: participants were health care providers working with older adults in LTC, the context was LTC, and the concept was AI-enabled robots. The search string was based on these participants, context, and concept, that is, “healthcare provider” AND “older adult” AND “LTC” AND “AI.” Our search was limited to the items published in the last 10 years (2013-2023), and we only included publications in English.

### Item Selection

We used a web-based software platform, Covidence (Veritas Health Innovation), to assist us in conducting the scoping review. Initially, we identified 279 items and uploaded them to Covidence. Subsequently, we performed 2 screening levels according to the inclusion and exclusion criteria (Textbox 1).

We adopted an inclusive approach to how we considered a robot an AI-enabled robot. We considered a robot an AI-enabled robot if it has AI features according to our AI-enabled robot definition as mentioned in the Introduction section. Textbox 2 explains how each robot included in this scoping review is related to our AI-enabled definition.

**Eligibility criteria.**

**Inclusion criteria**

- Includes health care providers working with older adults in long-term care (LTC) homes
- Includes artificial intelligence (AI)–enabled robots
- Includes LTC home setting
- Published in English language
- All study designs (eg, qualitative, quantitative, and mixed methods)
- All sources (eg, peer-reviewed journal articles, books and book chapters, conference proceedings, reports, theses, and dissertations)
- Include data that address the 2 objectives of our paper

**Exclusion criteria**

- Does not include health care providers working with older adults
- Does not include AI-enabled robots
- Acute care or community settings other than LTC home (eg, home care, older adult care centers, and adult day health care programs)
- Published in language other than English
- Do not include data that address the 2 objectives of our paper

**How robots are related to artificial intelligence (AI) by definition.**

**Robot name and how it is related to AI by definition**

- Stevie [48,49]
  Stevie is a social robot designed to be used in care settings for older adults. The robot incorporates AI to enhance its functionality and communication abilities. Like other AI-enabled robots, Stevie II can autonomously map and navigate 3D environments in real time. Unlike other robots, Stevie uses AI to support its enhanced communication functionality. Through the use of humanlike speech, gestures, and facial expressions, the robot is able to engage in clear communication required for social interaction. Furthermore, the robot leverages AI to perform various tasks such as setting reminders, entertaining older adults, and problem-solving.
- PARO [50]
  PARO is a seal-shaped interactive robot that incorporates sensory receptors to interface with its environment. The robot is used in various health care environments to reduce stress anxiety, improve socialization, and so on by replicating the effects of animal therapy. PARO can be held, spoken to, and stroked as if it were an actual animal. AI enables PARO to remember users’ actions and learn to adapt its behavior accordingly. Actions that result in positive user feedback will be repeated, while actions resulting in negative user feedback will not.
- NAO [51]
  NAO is a bipedal interactive humanoid robot designed for various applications, including education and research. The robot supports open-source functionality, allowing users to curate the robot to their specific needs. NAO uses AI at the lowest level to perceive the surrounding environment, understand and respond to human emotion, solve tasks, navigate using advanced vision systems, and so on.
- Pepper [52]
  Pepper is a humanoid robot designed for social interaction and customer service. Leveraging its ability to recognize faces and human emotions, Pepper is available in businesses and schools as a helpful assistant. The robot uses AI to map and navigate its environment and solve tasks, among many other things. Above all, Pepper uses AI to recognize and respond to human emotions, making it capable of humanlike communication. It also learns from interactions, allowing it to adapt and improve its responses over time.
- SCITOS [53]
  The SCITOS is an autonomous robot designed to be used in various applications such as research and many customer service positions. The robot is designed to interact with people through its voice, head movements, and touch display, as well as guide people in various settings. It uses AI to map and navigate its environment, avoiding obstacles and identifying its position in a 3-D space. AI is also used for effective communication, equipping SCITOS with the ability to interact with and understand humans. This AI-enabled emotional recognition allows the robot to guide visitors, provide explanations, and play fun games such as hide and seek.
- Tangy [54]
  Tangy is a social robot designed to assist with social and interactive tasks. The main use case for Tangy is as a bingo assistant in long-term care settings. Using AI, Tangy can autonomously support older adults in playing bingo. The robot is able to call bingo numbers, help individuals, ensure the accuracy of winning bingo cards, and congratulate winners. These functionalities are supported by AI, which enables Tangy to perceive and influence its surrounding environment. AI also allows the robot to recognize certain bingo cards as winners, an ability made possible with improved computer vision systems. Tangy also uses AI to integrate information from different sensors, and this allows it to identify and communicate when certain bingo numbers are called out.
- MARIO [55,56]
  The MARIO robot is a social robot that builds on existing Kompai architecture with the purpose of providing companionship and support. The robot is intended for use with individuals who have dementia in long-term care settings. The robot applies AI at various levels to supplement its predetermined functionalities. Simultaneous mapping and navigation are possible through the AI-driven integration of sensor data, although this requires more constant environments. It is also able to understand and respond to human communication, and this allows it to effectively engage with residents. The robot supports various applications, including fall or hazard detection, which are facilitated by AI.
- Roomba [57]
  The Roomba is a robotic vacuum cleaner designed to automate the floor cleaning process. There are several iterations of the device that support different types of cleaning and more efficient automation. At the lowest level, the Roomba uses AI to perceive and influence its surrounding environment. Advanced sensor and mapping technology is leveraged to create a computer-readable rendition of a 3D space, enabling systematic and efficient cleaning patterns.
- TUG [58]
  The TUG autonomous mobile robot is made specifically to deliver linens, medications, and meals in hospital settings. The robot functions to maintain order in the hospital and reduce the physical workload that the staff are required to manage. The TUG robot uses AI to enhance its computer vision capabilities, allowing it to map and navigate 3D environments and make use of existing navigation infrastructures (eg, elevator). The AI-enabled robot also can identify hazards in real time, which is integral in health care settings that may contain patients, caregivers, and other individuals.
- PaPeRo [59]
  PaPeRo is a social robot that was developed to appeal to various populations, with the intention of providing companionship. The robot uses AI to engage in human conversation, recognizing >200 words and speaking in a natural voice. The robot can also perceive the volume of sounds in the environment and adjust its behavior accordingly. Furthermore, the robot makes use of image-recognition technology to identify faces. While not actively engaging with individuals, the robot makes use of AI to map and traverse through its environment while dancing and singing on its own. PaPeRo is also able to identify hazards in its environment.
- Temi [60]
  Temi is a social robot designed for home and business applications. The robot acts as an autonomous personal assistant following users, saving locations, setting tasks, and providing Face Time functionalities. Temi uses AI to map and navigate its environment, facilitating autonomous motion. The robot can also identify environmental hazards and adapt its movement to suit different types of surfaces to suit different types of surfaces (wood, carpet, etc). It can also understand and respond to voice commands, demonstrating an ability to engage in humanlike communication while also learning and adapting to users’ behaviors over time.
- Grace [61]
  Grace is an advanced humanoid robot designed for health care applications. The robot emulates health care professionals with a humanlike appearance and its ability to interact with patients, record key vitals (temperature and responsiveness), engage in therapeutic conversation, and help other health care professionals. AI is deeply embedded into almost all components of Grace, allowing for autonomous movement, diagnosing of some conditions, and most of all, humanlike communication. The robot uses AI in conjunction with electrical components to replicate facial expressions, and this facilitate its ability to communicate and provide companionship. The robot is mainly used to provide social stimulation to older adults and others isolated in health care settings.
- PR2 [62]
  PR2 is a service robot that supports a wide array of use cases and can be adapted to fit different environments. PR2 robots have the dexterity to fold towels, grab drinks, pick up various items, and even make purchases in stores. The robot relies heavily on AI to perform these tasks. A variety of sensors are integrated through AI tools to create 3D representations of environments, allowing for real-time mapping and navigation. The robot also supports complex problem-solving skills that allow it to adapt to complete many different tasks. AI also enables the robot to interact with humans through voice and gesture recognition.
- Sota [63]
  Sota is a social robot optimized for social interaction and communication. Sota is designed to give PowerPoint presentations, making use of its versatile communication skills. The robot has a unique communication style, making use of its tone of voice, arm gestures, expressions, body language, and other sounds to convey certain emotions. Sota uses AI to integrate its different skills in a coherent way suitable for presentations. For example, the 3D environment is mapped in real time, and this allows Sota to move around while speaking and point to key features in the presentation. Sota’s voice and gestures are all decided on using AI algorithms that can learn and adapt to audience reactions over time.
- Smart Walker [64]
  The Smart Walker is an AI-assistive device that is intended to aid individuals with mobility challenges. The robot can precisely detect users’ movements and adjust its own behavior accordingly by moving forward, stopping, or adjusting its speed. AI allows the robot to map and navigate its environment while also detecting hazards in real time. The hazard detection functionality uses AI to detect objects in the path of the user and stop the walker, preventing collisions. Furthermore, the robot also supports gait detection and health monitoring, a feature enabled by the integration of various sensor data through AI.
- Artificial Intelligence Lightweight Android (AILA) [65]
  AILA is an autonomous robot designed as a research platform for autonomous mobile dual-arm manipulation. The robot can understand and navigate its environment in real time, a feature facilitated by integrating AI technologies with various sensor modalities. Moreover, AI enables AILA to recognize specific objects in its environment through feature matching and 3D pose estimation. On the basis of this information, AILA can autonomously adjust its arms and determine the best orientation to grasp different objects. Furthermore, AILA can use different strategies to lift and relocate objects depending on their characteristics (fragile, soft, and hard).

**Figure 2:**
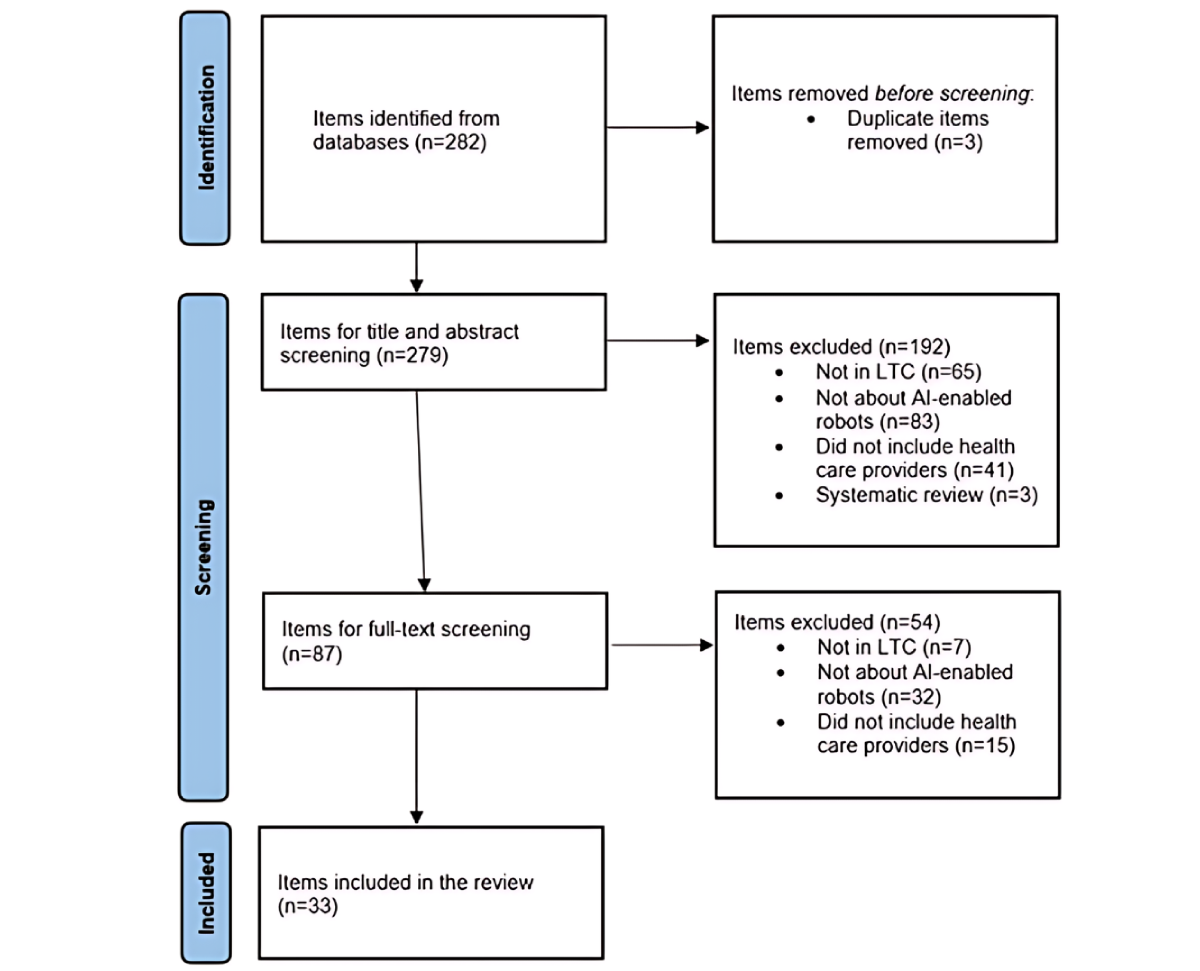
PRISMA (Preferred Reporting Items for Systematic Reviews and Meta-Analyses) chart. AI: artificial intelligence; LTC: long-term care.

In the first screening level, 2 research trainees of our team independently screened the titles and abstracts of the 279 identified items. We removed 192 (68.8%) items that were not conducted in LTC homes, were not about AI-enabled robots, did not include health care providers, or were systematic reviews, and 87 (31.2%) items remained. In the second screening level, the 2 research trainees independently reviewed the full text of the 87 selected items. Subsequently, we removed 54 items (reasons for removing were the same as those in the first screening level), and 33 items remained. We used the PRISMA (Preferred Reporting Items for Systematic Reviews and Meta-Analyses) statement [66] to record the selection process (Figure 2). When the 2 research trainees did not agree with each other, the researcher (LH) made the final decision.

### Data Extraction and Analysis

We extracted data from the chosen items by domain and documented the data extracted in the data extraction table (Multimedia Appendix 1 [11,49,67-97]). The domain included “author, year, and place” of the literature; “literature type and study design/method”; “setting, population, and sample size (if mentioned)” of the study; “type of AI-enabled robot and use of the robot” mentioned in the literature; “barriers to the use of the AI-enabled robot (from healthcare providers’ perspectives)”; and “strategies to overcome the barriers.” The data extraction tool was an enhanced version compared with the one we published in the protocol: most of the categories of the tool in the enhanced version were similar to the protocol version. The main difference was that we removed the category on “results and implications” and added a category on “the type of AI-enabled robot and use of the robot.” We initially had a category on “results and implications,” just in case we needed additional information to help us address the review questions. However, after we started data extraction, we realized that the category on “results and implications” did not provide additional information to help us address the review questions and might even divert our attention away from addressing the review questions. Therefore, after discussion, we removed this category. By contrast, during data extraction, we found that the differences in the types and uses of AI-enabled robots might relate to the review questions on the barriers and the strategies to overcome them. Therefore, after discussion, we added this category.

Each team member was randomly assigned items for data extraction. Since our older adult partner expressed interest in helping with data extraction despite being less familiar with academic work, he was assigned fewer items (3 items) than other members. We met to discuss the challenges we encountered during the data extraction process and resolve them together under the guidance of the researcher (LH), who is experienced in the scoping review. For items that include both health care provider participants and non–health care provider participants (eg, residents and families), we only extracted the data on the barriers and the strategies to overcome these barriers from the perspectives of health care providers. For items that include AI-enabled and non–AI-enabled robots, we only extracted the data on AI-enabled robots.

After data extraction, a research trainee of our team did a preliminary thematic analysis of the extracted data using NVivo (version 12; Lumivero), a qualitative data analysis software [98]. The trainee coded the data, grouped similar codes into categories, grouped similar categories into themes, and then presented the findings to the team. The team members gave inputs to refine the findings, which were finalized through team analysis. When differing opinions on the themes arose, we reached a consensus through discussion. Team members then compared the findings with PCPF and CFIR to see how the frameworks further our understanding of the findings. We followed a mixed inductive and deductive approach in data analysis. The research trainees coded the data, and the team developed the themes inductively. However, we also compared the findings with PCPF and CFIR to further our understanding of the findings deductively.

## Results

### Overview

In our review, we included 33 studies conducted across 16 different countries. Most studies were from Canada and Australia, with 12% (5/33) of the studies from each country [11,72,78,80,84,85,90,94,96]. Moreover, 9% (3/33) of the studies were conducted in Austria, and the United States [49,68,75,89,91,95]. There were 4 (12%) studies that took place in Japan [74,86,93,97]. The remaining countries contributed to either 3% (1/33) of the studies (China, the Netherlands, New Zealand, Turkey, and the United Kingdom) or 6% (2/33) of the studies (Finland, Germany, Switzerland, and Taiwan), while 1 (3%) [67,69-71,73,81,82,87-89,92] study involved >1 country (Italy, Ireland, and the United Kingdom) [81]. Most of the studies (20/33, 61%) were journal articles [11,49,67-72,74-77,79,80,82,83,86,87,92,97]. Furthermore, 24% (8/33) of the studies were conference proceedings, and 9% (3/33) of the studies were book chapters [73,78,81,84,85,88,89,91,93,95,96]. There was 1 (3%) web-based news article and 1 (3%) dissertation [90,94]. The most frequently researched robot among the studies was the social robot PARO, mentioned in 33% (11/33) of the studies [11,69,73,77,80,81,84-86,89,96]. The humanoid robots NAO and Pepper were mentioned by 15% (5/33) of the studies and 12% (4/33) of the studies, respectively [67,70-72,79,84,86,97]. While 2 (6%) studies were found mentioning the following robots: SCITOS, Sota, and Tangy, 1 (3%) study mentioned the remaining robots [49,68,73-76,78,83,87,88,90,91,93,94].

### Barriers to the Use of AI-Enabled Robots

#### Overview

One of our primary research questions is to explore the barriers that health care providers perceive in adopting AI-enabled robots in LTC homes. After reviewing and analyzing the existing literature, 3 barriers related to this regard were identified (Textbox 3).

**A summary of the barriers to the use of artificial intelligence (AI)–enabled robots.**

**Barriers and summary**

- Perceived technical complexity and limitation
  Perceived using AI-enabled robots complex or troublesome
  Reported not having the knowledge and skills to use the robots
- Negative impact, doubted usefulness, and ethical concerns
  Worried about the AI-enabled robots’ negative impacts on residents
  Worried about the AI-enabled robots’ negative impacts on staff
  Doubted residents’ interests in the AI-enabled robots
  Doubted the usefulness of the AI-enabled robots to residents
  Doubted that the robots would fit the LTC home context
  Worried about the potential ethical issues of using AI-enabled robots
  Raised ethical concerns on privacy
- Resource limitations
  Lack of human and time resources
  Not enough robots
  A lack of infrastructure
  Worried about maintenance costs

#### Perceived Technical Complexity and Limitation

Health care providers perceived the technical complexity and limitations of robots as a barrier to adopting robots. For example, in the study by Huisman and Kort [67], using the humanoid robot NAO to entertain residents and stimulate them to do physical exercises, health care providers expressed frustration with the complex robot’s operation steps and the robot’s short battery life. In the study by Hebesberger et al [68], the autonomous robot SCITOS was used to perform safety checks around the LTC home (eg, checking if doors were closed and fire extinguishers were in place) and greet visitors at the LTC home’s lobby. Nevertheless, health care providers reported that the robot’s slow response and rigid system discouraged their use of it. In the study by Pfadenhauer and Dukat [69] on the social robot PARO, health care providers mentioned that as the robot could not move independently, they had to carry it around the LTC home for residents’ use, which was inconvenient.

In addition, a lack of knowledge and skills contributes to the reluctance to adopt these technologies. For example, in the study by Papadopoulos et al [70], using the humanoid robot Pepper to interact with residents socially, health care providers reported not being fully equipped to operate and maintain the robot. In the study by Melka et al [71], using the humanoid robot NAO for rehabilitation and recreational assistance, health care providers hesitated to use the robot as they feared making mistakes because of a lack of knowledge and skills using it. Even if there is training, the willingness to participate is low due to existing heavy workloads. For instance, referring to the study by Li et al [72], using humanoid robots Pepper and NAO to entertain residents, health care providers expressed concerns about the time needed to join training as they already had heavy workloads.

#### Negative Impact, Doubted Usefulness, and Ethical Concerns

Health care providers have expressed concerns, including potential negative impacts, doubts about their usefulness, and ethical implications.

#### Potential Negative Impacts

Some health care providers are concerned that AI-enabled robots might negatively impact residents. For example, one of the robots used in the ethnographic study by Chang and Šabanović [73] was the robotic vacuum Roomba, and health care providers expressed concerns about residents’ risk of falling. In the study by McGinn et al [49], using the social robot Stevie to communicate with residents, health care providers were worried about hygiene hazards, such as the robot not being adequately sterilized after use and the potential spread of germs among residents. The study by Obayashi et al [74] used the social robot Sota; The health care providers were concerned that the robot’s flashing eyes might scare residents.

Some health care providers were concerned that the AI-enabled robots might negatively impact them. For example, in the study by Hung et al [11], on the use of robots, including social robot PARO, in LTC, health care providers expressed their concerns that using these robots increased their workloads, such as teaching and assisting residents to use the robots, cleaning and charging the robots, and handling technical glitches. The study by Mitzner et al [75] used service robot PR2 to assist in caregiving tasks such as medication dispensing and transferring residents. Health care providers were concerned that the robot would replace human functions. In the study by Melkas et al [71], using the humanoid robot NAO to assist in rehabilitation and recreation, health care providers were reluctant to use it as they had established workflows, and including the robot would interrupt how they had been doing things. Erebak and Turgut [76] studied health care providers’ attitudes toward robots, such as the autonomous robot Artificial Intelligence Lightweight Android (AILA). They found that the health care providers did not trust robots to be fully autonomous in decision-making. Instead, they preferred robots that allowed them to make certain decisions as humans (although the authors did not specify what these decisions would be).

#### Doubted Usefulness

Some health care providers have raised doubts about the usefulness of AI-enabled robots for residents in LTC homes. Some providers doubted whether residents would be interested in robots. For example, in the study by Robinson et al [77], on social robotic pet PARO as a companion with residents, health care providers raised doubts that residents would be interested in it because residents preferred real pets, and PARO looked like a toy. In the study by Louie et al [78], using the social robot Tangy, health care providers said residents preferred humans, and Tangy’s appearance and voice were too mechanical. In the study by Huisman and Kort [67], using the humanoid robot NAO, health care providers said that residents would be bored by the lack of choices of programs that the robots could offer for recreation and physical exercise.

Other health care providers doubted the practicality of AI-enabled robots for residents. For instance, in the study by Louie et al [78], the primary function of the social robot Tangy was to speak to residents and interact with them. However, Tangy could not speak the languages of some residents. Some health care providers pointed out that these residents could not understand and interact with Tangy. The study by Bäck et al [79] used humanoid robot NAO to demonstrate physical exercise to residents. However, health care providers hesitated to use the robot because its size was too small for residents with eyesight impairment to see it, its voice was too soft for residents with hearing impairment to hear its instructions, and residents with cognitive impairment could not follow its demonstration.

Furthermore, providers doubted that the robots would fit the LTC home context. For instance, one robot used in the study by Chang and Šabanović [73] was the autonomous mobile robot, TUG, which was designed to help health care providers deliver care and medical supplies and clean the LTC home. However, health care providers did not find it helpful because its size was too large to navigate the LTC home’s narrow hallways.

#### Potential Ethical Issues

Health care providers have expressed ethical concerns about using AI-enabled robots in LTC settings. For example, in the study by Moyle et al [80], social robot PARO was used to interact with residents. Health care providers raised concerns that the use might infantilize residents due to the robot’s toy-like appearance. In the study by Lehmann et al [81], PARO was used as a robotic pet for companionship with residents. Health care providers expressed concerns that the robot could deceive residents with cognitive impairment as a real pet.

Privacy concerns have also been raised, particularly concerning surveillance. For instance, Christoforou et al [82] looked into different types of nursing and social and physical assistive robots. Some health care providers expressed the feeling that the robots were monitoring their work. In the study by Papadopoulos et al [70], using the humanoid robot Pepper to communicate with residents, health care providers worried about residents’ privacy, especially since Pepper had cameras on its forehead.

#### Resource Limitations

The last barrier expressed by health care providers is that LTC homes lack the resources to use AI-enabled robots. The types of resources that LTC homes lack include time, robots, infrastructure, and maintenance costs. First, as suggested, health care providers expressed concerns about the lack of time to learn, maintain, and assist residents using the robots [11]. Second, some health care providers said that their LTC homes did not have sufficient robots. In the study by Hung et al [11], a nurse recalled how 2 residents fought with each other for 1 social robot PARO. Third, some health care providers mentioned that their LTC homes lacked infrastructure for robot use. Melkas et al [71] used a humanoid robot NAO for diverse purposes (eg, rehabilitation and recreation), and NAO needed Wi-Fi. However, some health care providers mentioned that their LTC home had a poor internet connection. They also added that their LTC home did not have sufficient physical space to store the robot. Finally, keeping a robot is expensive, and some health care providers were worried about the maintenance costs. In the study by Casey et al [83], using the social robot MARIO to stimulate residents’ cognition and memories, such as giving them updates on news, health care providers were concerned about the costs of keeping the robot and suggested that it would be a better idea to spend money hiring more health care providers than keeping the robot.

### Strategies to Overcome the Barriers to the Use of AI-Enabled Robots

#### Overview

To overcome the barriers, another primary question of our review is to identify the strategies suggested in the literature, including (1) accommodate the various needs of residents and health care providers, (2) increase the understanding of the benefits of using robots, (3) review and overcome the safety issues, and (4) boost interests in the use of robots and provide training. The strategies can be summarized in an acronym, “AI-ROBOT” (Textbox 4).

**Summary of the strategies to overcome barriers.**

**AI-ROBOT and illustrative examples from literature**

- Accommodate various needs of residents and health care providers
  Incorporate songs in languages other than English to meet the language needs of residents [84]
- Increase the understanding of the benefits of using robots
  Health care staff found that the robot could enhance residents’ emotional well-being and bring them joy [67]
- Review and overcome the safety issues with staff
  Incorporate safety designs suggested by staff into robots [88]
- Boost interests in the use of robots
  Dress up the robots to make them more attractive [90]
- Provide training and involve staff in the planning and implementation
  Set up a help desk for health care staff to contact by phone or email when they encounter any challenge using the robot [67]

#### Accommodate the Various Needs of Residents and Health Care Providers

One strategy is to collect feedback from health care providers to design AI-enabled robots that better accommodate the various needs of residents and health care providers. For example, one of the robots used in the study by Yuan et al [84] was the humanoid robot NAO for communication with residents. Health care providers raised a concern that some residents could not understand English. In response to this feedback, Yuan et al [84] suggested incorporating songs in languages other than English into the robot to meet these residents’ language needs better. The study by Bäck et al [79] used NAO for demonstration of physical exercise. In response to the feedback from health care providers that residents with sight impairment could hardly see the robot and the residents with hearing impairment could not hear the instructions by the robot clearly, Bäck et al [79] recommended painting the arms of the robot in sharp color and giving it a loud and clear voice to accommodate the visual and hearing needs. In the study by Cavenett et al [85] who used the social robot PARO for social interaction with residents, corresponding to health care providers’ concerns that using the robot would add to their workload and interrupt their established workflow, Cavenett et al [85] proposed acknowledging the concerns and discussing with health care providers to understand their work needs and explore how the use of robot could address these needs and fit with their existing workflow.

#### Increase the Understanding of the Benefits of Using Robots

Another strategy is to increase health care providers’ understanding of the benefits of using robots. The authors of the literature reviewed mentioned that when health care providers better understand the benefits of using robots, they will accept and use them more. For example, in the study by Huisman and Kort [67], some health care providers supported using the humanoid robot NAO because they found that it could enhance the emotional well-being of residents and bring them joy. In the study by Kolstad et al [86], some health care providers welcomed the social robot PARO because they found that it could stimulate residents’ functions, such as interactions with people. In the study by Follmann et al [87], the social robot Temi was used to contact residents’ relatives. Health care providers welcomed the robot because they found different benefits to it: it was easy for residents to use and health care providers did not need to transport the robots between residents, did not need to stand by to provide supervision when residents were using the robot, and did not need to disinfect the robot.

In addition, the reviewed literature suggests letting health care providers learn that the relationship between health care providers and robots is not competitive but complementary, emphasizing that humans perform certain tasks better than robots. A collaborative approach leverages the strengths of robots and humans, ensuring a higher quality of care. For example, Cavenett et al [85] used social robots to communicate with residents. Corresponding to health care providers’ worry that robots would replace their communication role, the authors suggested that robots only complemented health care providers’ communication role because robots were not as capable as humans to catch the nonverbal cues of residents.

#### Review and Overcome the Safety Issues With Staff

The third strategy is to review the safety issues with staff and address their concerns. The study by Shin et al [88] used the robot SmartWalker to guide residents to walk. The health care providers asked if the robot was safe, considering that the residents were prone to falling. Shin et al [88] recommended incorporating safety designs into the robot. For example, when there were obstacles and stairs in front of residents, the robot would give audio warnings to the residents. In the study by Hung et al [11], in response to health care providers’ concerns about residents’ safety that residents might fight over the robots, Hung et al [11] proposed having risk assessment and management guidelines in place to avoid conflicts and violence over robots and guide health care providers on what to do in case these happen.

#### Boost Interests in the Use of Robots

It is crucial to boost interests in using the AI-enabled robots from both the health care providers and the residents. The authors of the reviewed literature suggested increasing health care providers’ interest in using robots. For instance, in the study by Chang and Šabanović [89], the researchers used the social robot PARO with residents in public areas of LTC so that health care providers could see the process of using the robot and witness how the robot provided therapeutic effects to residents. The researchers found that this raised the health care providers’ interest in using the robot because they told the researchers that seeing how the researchers used the robot with the residents stimulated them to think about how they could use it in their work.

Since health care providers expressed concerns that residents might not be interested in using the robot, the authors of the reviewed literature also suggested increasing residents’ interest in using robots to address their concerns. Robinson et al [77], who used the social robot PARO, proposed changing the robot’s color from white to a more appealing color. Louie [90] recommended dressing up the social robot Tangy so that it looked more attractive. In the study by Hebesberger et al [91], using the robot SCITOS to accompany residents doing walking exercises, the researchers suggested giving the robot a name so that residents felt the robot was more personalized and thus more interested in it. In addition, in response to health care providers’ comments that SCITOS’s voice was too mechanical, they proposed giving the robot a more attractive, more natural voice.

#### Provide Training and Involve Staff in the Planning and Implementation

The last strategy is to provide training. The authors of the reviewed literature recommended training and support to health care providers. Huisman and Kort [67], who used the humanoid robot NAO, proposed that the training should give health care providers clear instructions on how to use the robot for physical and recreational activities with residents. They added that time needed to be reserved for training. Otherwise, health care providers could not find time to do the training within their busy work schedules. They also recommended setting up a help desk, which health care providers could contact by phone or email when they encountered any challenges using the robot. When health care providers were more familiar with using the robot, they recommended peer learning (ie, encouraging the health care providers to support each other in using the robot). Yuan et al [84], who used the social robot PARO and humanoid robot NAO for interaction and communication with residents, raised the need for an instruction manual in place so that health care providers could refer to it after training.

### Comparing Findings With Theoretical Frameworks

#### Overview

As suggested, we compared our findings with PCPF and CFIR to further our understanding of the findings. The authors of PCPF [40] and CFIR [99] suggested that discussing all constructs in 1 paper is not feasible, so they recommended that the users of their framework select a few most relevant constructs to the research. We selected constructs most relevant to our review through team discussions.

#### Comparing Findings With PCPF

In our review, one barrier to adopting AI-enabled robots is that health care providers feel that they lack the knowledge and skills to use the robots [70]. One strategy is providing training to health care providers so that they can improve their knowledge and skills [67]. The knowledge and skills needed to use robots to provide care may be part of “professional competence,” a construct under the “prerequisite” domain of PCPF. This construct refers to “the knowledge, skills and attitudes of the person to negotiate care options and effectively provide holistic care” [36].

Another barrier mentioned in our review is that health care providers are concerned that robots might replace human functions [75]. One strategy is to let health care providers know that they are better at providing these human functions, such as communication, than robots, as they are humans [75]. The constructs “engaging authentically” and “being sympathetically present” under the “person-centered process” domains of PCPF help us further understand why health care providers are better than robots. “Engaging authentically” refers to “the connectedness between people, determined by knowledge of the person, clarity of beliefs and values, knowledge of self and professional expertise” [22]. Robots cannot engage with residents “authentically” because robots are not authentic humans, despite being equipped with AI. “Being sympathetically present” means “an engagement that recognizes the uniqueness and value of the patient by appropriately responding to cues that maximize coping resources through the recognition of important agendas in the person’s life” [36]. The study by Cavenett et al [85] in our review mentioned that humans are better than robots at responding to nonverbal “cues” of residents, which are a crucial element to show residents “being sympathetically present.”

The “shared decision-making” construct under the “person-centered process” domain of PCPF refers to “engaging persons in decision-making by considering values, experiences, concerns and future aspirations” [36]. This construct made us wonder why health care providers did not mention too much about how the lack of residents’ involvement in decision-making was a barrier to the adoption of robots or suggest involving residents’ voices in the adoption process, especially since many barriers that the health care providers mentioned were related to the residents, such as potential negative impacts of robots to residents [49,73,74], perceived lack of usefulness of robots to residents [67,77,78], and ethical concerns related to residents [70,80,81]. One explanation might be that health care providers thought that they knew the residents well and could represent the voices of residents, so involving residents in decision-making was not a concern from their perspective. Future studies might examine this further. Another construct is “health and social care/policy” under the “macrocontext” domain of PCPF, which refers to “the decisions, plans, and actions that are undertaken to achieve specific health and social care goals within a society” [36]. Health care providers in our review did not mention too much about the influence of health and social policy on their adoption of AI-enabled robots, although there were international and national policies about the use of AI, such as the guidelines by the World Health Organization [100] and Health Canada [101].

#### Comparing Findings With CFIR

One barrier to adopting the AI-enabled robots mentioned in our review is health care providers’ perceived technical complexity [68]. CFIR has a construct of “innovation complexity” under the “innovation” domain, which resonated with our findings. This construct states that “the innovation is complicated, which may be reflected by its scope and/or the nature and number of connections and steps” [37].

Another barrier is a lack of resources, such as time for health care providers to learn, maintain, and assist residents in using robots [11]; infrastructure such as Wi-Fi and storage place [71]; and maintenance costs [83]. CFIR has the construct “available resources” under the “inner setting” domain [37]. “Available resources” means “resources (that) are available to implement and deliver the innovation” [37]. This construct helps us think that the lack of resources is an organizational-level barrier, as the “inner setting” in our review context means the LTC home (ie, the organization). In other words, the organization needs to be involved in resolving these barriers.

The construct “need” is under the “individuals” domain of CFIR [37]. The “need” construct refers to the following: “the individual(s) has deficits related to survival, well-being, or personal fulfillment, which will be addressed by implementation and/or delivery of the innovation” [37]. Our findings include barriers such as health care providers’ doubted usefulness [79] and ethical concerns [81] about using robots for residents. The “need” construct helps us realize that these barriers are related to health care providers’ concerns about the “needs” of residents. For example, in the study by Bäck et al [79], health care providers doubted the usefulness of the humanoid robot NAO in facilitating exercises because its size was too small for residents with visual impairment to see it, and its voice was too soft for residents with hearing impairment to hear it. It does not meet the visual and hearing “needs” of residents. In the study by Papadopoulos et al [70] on the humanoid robot Pepper, health care providers were worried about residents’ privacy as the robot had a camera on its forehead: they were concerned about the privacy “need” of residents.

In our review, some health care providers hesitated to use robots because they were concerned that doing so would interrupt their existing workflow [71]. One strategy identified was to discuss with health care providers how to integrate the robot into their workflow [85]. The construct “compatibility” under the “inner setting” domain of CFIR helps us to understand that these are “compatibility” concerns [37]. “Compatibility” refers to the following: “the innovation fits with workflows, systems, and processes” [37].

## Discussion

### Principal Findings

This scoping review addressed two review questions: (1) health care providers’ perceived barriers to using AI-enabled robots in LTC and (2) the strategies to overcome the barriers to the adoption of robots. We identified the barriers to adopting AI-enabled robots in LTC homes, including (1) perceived technical complexity and limitation; (2) negative impact, doubtfulness, and ethical concerns; and (3) resource limitations. We also identified the strategies to overcome these barriers to adopting AI-enabled robots: (1) accommodate various needs of residents and health care providers, (2) increase the understanding of the benefits of using robots, (3) review and overcome the safety issues, and (4) boost interest in the use of robots and provide training.

As suggested in the Introduction section, LTC homes face the challenges of increased resident care needs and a shortage of health care providers [3-5]. Researchers and technology developers expect that AI-enabled robots could address these challenges by sharing the workload of health care providers [6-8]. However, according to the results of our review, health care providers had a different opinion. For example, some suggested that using robots would increase instead of reducing their workload, such as assisting residents in using the robots and cleaning them [11]. Some suggested that using robots would interrupt their existing workflow [71]. An explanation for the gap between researchers or technology developers’ expectations and health care providers’ opinions might be health care providers’ lack of involvement in developing and researching AI-enabled robots. Therefore, health care providers’ opinions did not align with researchers’ and technology developers’ expectations that robots could address the challenges in LTC homes.

In this scoping review, health care providers identified a few ethical concerns about using AI-enabled robots. Frennert and Östlund [102] summarized the main ethical concerns of using robots with older adults from previous literature. Although the literature summarized by Frennert and Östlund [102] did not specifically address AI-enabled robots or LTC contexts, it could still be a good reference for understanding the findings on ethical concerns in our review. Some ethical concerns mentioned in the paper by Frennert and Östlund [102] were found in our review, including concerns about the privacy of residents [70] and health care providers [82]; deception to residents [81]; infantilization of residents (as many robots, especially social robots, look like a toy) [80]; equitable distribution of the use of robots in a group setting [11]; and residents’ attachment to the robots and negative emotions triggered when they break down [70]. However, when Frennert and Östlund [102] mentioned the ethical concern of humans’ loss of control of the robot, they did not specify the particular aspects of control involved. In our scoping review, for example, in the study by Erebak and Turgut [76], health care providers mentioned specifically the control of decision-making. One possible reason health care providers in our scoping review highlighted the control of decision-making is that among different aspects of control of AI-enabled robots, decision-making is a key feature of AI that is widely discussed in society [103].

In our review, health care providers had different opinions on whether the AI-enabled robots should look real. Some providers suggested that residents preferred real people or pets and doubted that residents would be interested in robots if they were not real enough [77,78]. Corresponding to this feedback, some literature proposed making the robot more real by adding features (eg, a more natural human voice) [91]. However, some health care providers had an ethical concern that residents with cognitive impairment would be deceived that the robots were real [81]. The concern about deception was also previously discussed in the literature on non–AI-enabled robots. For example, in the study by Koh et al [104], health care providers raised their concern that residents misperceived a Joy for All companion cat as a real cat. We considered that the opinion divide would be even more intense as AI-enabled robots develop. Compared with non–AI-enabled robots, AI-enabled robots could be even more “real” due to AI technology. Future research might further explore this topic.

### Recommendations

As suggested, a possible explanation for the gap between researchers or technology developers’ expectations and health care providers’ opinions on using AI-enabled robots is health care providers’ lack of involvement in developing and researching AI-enabled robots. Previous literature on using technologies in older adults care highlighted the importance of involving health care providers [105]: working on the frontline, health care providers use technologies according to the context. Their experiences with technologies may differ from what researchers and technology developers expect. Thus, researchers and technology developers need to work with health care providers to understand, for example, their training requirements, how technologies can support their work, and how these technologies can be integrated into their established work routines and workflows. Involving health care providers and understanding their perspectives should increase their acceptability and sense of ownership and reduce their concerns about using technologies.

We propose that administrators be involved in addition to health care providers. Some barriers to using AI-enabled robots found in this scoping review could not be resolved without administrators’ involvement. For example, health care providers said that they did not have time for training [72], and the administrators are responsible for reserving time for them for training. Some health care providers are worried about the safety risks of using robots [49,73]. Administrators need to implement protocols for assessing and managing the risks.

We propose that residents be involved in addition to health care providers and administrators. When we compared our findings with PCPF, it was interesting that the health care providers in our review did not mention much about residents’ involvement, although they provided person-centered care. Residents are the users of the robots, and they can give feedback on using them.

All stakeholders, researchers, technology developers, health care providers, administrators, and residents need to get involved in developing and implementing AI-enabled robots. Each stakeholder has strengths to contribute: health care providers know the potential day-to-day challenges of using robots as they work on the front line, while administrators know the policies and regulations of LTC, so they can advise on how to ensure that the use of robots aligns with these policies and regulations. Technology developers have technical knowledge about robots, and residents provide feedback as users of the robots. This scoping review explored the barriers and the strategies to overcome the barriers to the use of robots from the perspectives of health care providers. Future scoping reviewers may consider conducting scoping reviews on other stakeholders’ perspectives.

### Limitations of the Review

This scoping review included only English-language studies because of the limited language capacity of our team. However, in many parts of the world, there is rapid development in the use of AI-enabled robots in LTC homes. Relevant studies published in languages other than English may have been omitted. Future scoping reviews should consider how to search for and include items in languages other than English, such as including team members with language capacity other than English or using translation tools. Furthermore, a quality assessment of the included studies was not conducted. However, the scoping review methodology mainly focuses on identifying the breadth of existing research rather than evaluating the quality of evidence [106]. Finally, the review only examined the point of view of health care providers, not residents. Future researchers may consider conducting a scoping review on the same topic but from residents’ perspectives.

### Conclusions

This scoping review examined the barriers to using AI-enabled robots in LTC homes from health care providers’ perspectives and identified the strategies to overcome these barriers to the adoption of such robots. We anchored our analysis in established theories, specifically the PCPF and the CFIR, to guide our further understanding of the findings. By addressing the barriers and identifying the strategies to overcome them, we hope to foster the effective deployment of AI-enabled robots in LTC homes.

## Appendix

Multimedia Appendix 1 Data extraction table.

Multimedia Appendix 2 Preferred Reporting Items for Systematic reviews and Meta-Analyses extension for Scoping Reviews (PRISMA-ScR) Checklist.

## Abbreviations

AI: artificial intelligence
AILA: Artificial Intelligence Lightweight Android
CFIR: Consolidated Framework for Implementation Research
LTC: long-term care
PCPF: Person-Centered Practice Framework
PRISMA: Preferred Reporting Items for Systematic Reviews and Meta-Analyses
