# Supplementary material for: Adoption of Artificial Intelligence–Enabled Robots in Long-Term Care Homes by Health Care Providers: Scoping Review
Source: JMIR Aging. 2024 Aug 27;7:e55257. doi: 10.2196/55257 (PMC11387915; doi:10.2196/55257)
Supplement: Multimedia Appendix 1 [file aging_v7i1e55257_app1.docx]

*Appendix 1: Data extraction table*

| **Author, Year, Place** | **Literature type and**  **Study Design/ Method** | **Setting, Population and Sample Size (if mentioned)** | **Type of AI-enabled Robot and Use of the Robot** | **Barriers to the use of AI-enabled robot**  **(from healthcare providers’ perspective)** | **Strategies to overcome the barriers** |
| --- | --- | --- | --- | --- | --- |
| Bäck et al., 2013, Finland [79] | - Journal article - Interview, survey | - Nursing home - 24 residents, 13 healthcare providers | - Compact humanoid robot NAO - Demonstration of physical exercise | - Healthcare providers had reservations about using the robot for residents with later stages of cognitive impairment and/or certain physical impairments (e.g., sight impairment, hard of hearing) as these residents might not be able to follow the exercise cues of the robot | - Robots adjusted so that its cues to residents on exercise were loud and clear, which helped residents with later stages of cognitive impairment to follow - Arms of the robot were painted in sharp colour, which helped residents with sight impairment to follow - Healthcare providers present to assist residents if needed |
| Casey et al., 2020, United Kingdom, Italy and Ireland [83] | - Journal article - Interviews | - Nursing home, community - 38 people with dementia; 28 family carers; 28 healthcare providers and 13 managers | - Social Robot MARIO - Maintain memories through reminiscence; entertainment; give updates on the news and personal interests of the people with dementia; give reminders on events | Healthcare providers:   - hesitated to use robots as they worried that robots might replace human interactions - hesitated to adopt the robots as they thought that the robots were not useful for people with later stages of dementia who might find it challenging to interact with the robots (e.g., touching screens) unless there was support - doubted the capacity of robots to provide individualized responses, cues, and care to people with dementia - wondered if the money spent on robots should be spent on hiring more healthcare providers | - Robots should:   - include more features to better understand and communicate with people with dementia e.g., speech recognition   - incorporate more human-like features   - be easy for healthcare providers to use - More education for healthcare providers so that they can change their negative attitudes toward robots and be more prepared to work with technology |
| Cavenett et al., 2018, Australia [85] | - Conference proceeding - Interviews | - Residential aged care - 5 healthcare providers | - Social robots such as PARO - Social interaction | Healthcare providers:   - resisted the new technology as it intervened in the established routines - hesitated to join the training and use the robots - lacked the knowledge and skills to resolve minor tech problems without trainings - were discouraged from using the robot by technical problems | - Ensure that healthcare providers are involved in the whole process of using the robots - Ensure to have conversations with healthcare providers on how the robots can be incorporated into their routines - Acknowledge that introducing the robots would intervene in the healthcare providers’ current routine - Provide training to healthcare providers early - Clear information on the expectations of using the robots - Be transparent about the potential challenges of using the robots and explain their benefits during the robot introduction to healthcare providers - Be transparent about the time healthcare providers can spend on the robots (including maintenance and training) - Reassure healthcare providers that robots cannot replace human interactions |
| Chang and Šabanović, 2015, United States[89] | - Conference proceeding - Observational sessions; informal interviews with residents and frequent visitors; semi structured interviews with healthcare providers | - Nursing home - Residents, healthcare providers, visitors | - Social robots such as PARO - Social interaction | N.A. | Use the robot in public spaces with residents, so that healthcare providers can see the therapeutic effects of the robot on residents and encourage staff to use the robot in their work with residents |
| Chang and Šabanović, 2013, Taiwan [73] | - Conference proceeding - Observational field study; interviews; focus groups | - Nursing home - Managers, healthcare providers, family members | - Robotic vacuum Roomba, Autonomous mobile robot TUG, Social robot PaPeRo, Social robot Paro - Assist patients with daily tasks and social interactions | - Risks and concerns associated with robots, for example: - Roomba robots were seen as a potential cause of falls - The large size of the TUG robot was perceived as a hindrance, making it difficult to navigate through narrow hallways | - Robots should be used as assistants rather than functioning independently |
| Chen et al., 2019, Taiwan [92] | - Journal article - Cross sectional study; surveys | - Different settings including care homes - 416 of health professionals, care workers and management personnel | - Social robots, types of robots not specified - Social interaction | - Respondents had mixed views on whether social robots would be a threat to aged care services | N.A. |
| Christoforou et al., 2020, China [82] | - Journal article - Survey/ questionnaire | - Different settings, including care homes - 115 Nurses, researchers, university graduates | - Nursing robots, socially assistive robots, physically assistive robots - Nursing robots relieve burden from nurses allowing them to concentrate on tasks pertinent to their primary duties - Socially assistive robots for social interactions - Physically assistive robots provide assistance required to stand-up, sit and walk | - Robots would threaten healthcare providers job security - Robots deployed at home might replace personal contact and assistance, i.e. loss of companionship and increased isolation - Robots might violates privacy as they created the feeling of being under continuous surveillance | - Apply an ethical framework overseeing nursing robot operations |
| Doi et al., 2016, Japan[93] | - Book chapter - Assessment, questionnaire | - Nursing homes - 12 healthcare providers | - Social robot Sota - Gesticulate and speak words of starting recreations or encouragement | N.A. | The healthcare providers acknowledged and recognized that the robot shared their workload and saved their time, so that healthcare providers could spend more time with residents |
| Erebak and Turgut, 2018, Turkey[76] | - Journal article - Experimental study | - Nursing home - 102 formal caregivers | - Autonomous Robot AILA | N.A. | - Healthcare providers preferred a robot with less automation in decision-making functions - Healthcare providers preferred robots with a less human-like appearance |
| Follmann et al., 2021, Germany[87] | - Journal article - Questionnaire | - 2 nursing homes and 1 hospital - 70 residents, formal caregivers (number not specified) | - Social robot Temi - Communication/ to combat loneliness and social isolation | N.A. | Healthcare providers acknowledges advantages of Temi:   - can allow direct contact with relatives - is easier to use than a tablet - can drive autonomously into quarantine rooms - can be left alone with residents with no supervision needed. - is with a surface that can be disinfected - provides entertainments (e.g., music or videos) |
| Hebesberger et al., 2017, Austria [68] | - Journal article - Mixed-Method design; observations; interviews; questionnaire | - Care home - 8 different healthcare professionals were interviewed; 70 questionnaires received from employees | - Autonomous robot SCITOS - Autonomous navigation and patrolling to check if doors were closed as needed and if fire-extinguisher was in place, greeting task at lobby | Healthcare providers:   - did not know that they could use the robot - did not try to use the robot because it did not offer games - feared to damage the robot when using it - feared to make mistakes when using the robot - perceived that the robot might lead to irritation - were afraid that the robot would replace healthcare providers - became bored by the robot with its invariant programme, functionalities, and lack of interactivity were annoyed by the robot's voice output and repeated sentences with an artificial voice - found that robots had slow reaction times, little autonomy and navigation problems | Healthcare providers perceived that robots could:   - compensate human mistakes - complement and improve human abilities - conduct everyday tasks that may tire human healthcare providers - bring potential economic advantage (e.g., robots could work all around the clock and have low ongoing costs) - elicit a positive atmosphere and make employees smile, laugh when interacting with the robot - Healthcare providers wanted more ways to interact with the robot besides touchscreen, e.g., through interactive language communication - Healthcare provides wanted robots with more natural voice, and even with local dialects |
| Hebesberger et al., 2016, Austria [91] | - Conference proceeding - Mixed-methods study; observations; rating scales;  interviews | - Care hospital / care home - 10 residents being observed; 4 therapists participated in rating observation scale and interviews | - Autonomous robot SCITOS - As a companion for the walking groups in physical therapy with the following functions: - serving as a source of motivation - accompanying the walking group - supporting social interaction within the group by providing topics therapists and residents can talk about during the sessions - providing an acoustic stimulus with playing musical background for singing during the walking periods or playing specific natural sounds in predefined areas of the building (e.g., playing the sound of cow bells in a corridor with paintings showing cows in a field) - offering entertainment and activation for the participants during resting periods (i.e. music and a picture gallery) | Healthcare providers found that:   - Older adults were not familiar with using touchscreens - Older adults with dementia would not be able to manage the menu on their own - Not all residents can be engaged with the robot at once   Healthcare providers:   - Experienced technical difficulties (e.g., the music and picture galleries were not always accessible with unstable Wi-Fi connections, robots did not respond immediately to control-card) - Experienced excessive demand to handle the robot in case of technical problems - Found it tedious to adjust the music volume - Felt that there was a lack of flexibility in the system (e.g., adjustment of the robot speed and order of the songs) | Healthcare providers’ positive attitude towards the robot   - Robots were funny, cool, exciting, and had positive charisma for older adults - Robots were easy to handle - Appreciated that the robot recognizing them as reference persons - Robot was a useful entertainment tool - Therapist felt relieved they could hand over own entertainment activities to the robot - Older adults were encouraged to interact with robots with the facilitation by healthcare providers - Personalization of robots with names |
| Huisman and Kort, 2019, the Netherlands [67] | - Journal article - Evaluation Study; interviews; observation; questionnaire | - 14 nursing care organizations (15 locations) - Interviews: 15 management and Board and 20 professional carers  Questionnaires: 62 healthcare providers | - Humanoid robot Zora (NAO) - For pleasure and entertainment or to stimulate the physical activities of residents | - No collaboration for using Zora (NAO) - No support from colleagues - Healthcare providers were not aware that they were allowed to choose to work with the robot - Healthcare providers felt frustrated and disappointed as starting the robot took more time than expected   Technical issues with the robot:   - Short battery life - Poor listening proficiency and speech skills (speech is unintelligible, responses from elderly clients are misunderstood, leading to incorrect responses) - Too complicated to program activities - Few pre-programmed activities available - Communicating through the robot was difficult because healthcare providers have to type the words on the composer at the same time | - Improved short-term Wi-Fi access in the buildings - Included an implementation for future improvements of the ICT (Information and Communications Technology) infrastructures in the organization’s agenda - Knowledge shared between professionals about using social robots, the tips for using the robot, and working with the robot in daily routines - Available instruction training and the Helpdesk by phone or email - Positive attitudes from healthcare providers: they felt more fun at work and happy when working with the robot; received enough time to learn how to work with the robot; perceived that the support to use the robot was sufficient and aligned with healthcare providers’ needs; believed that residents were content when the robot was used; preprogrammed dances and games were funny for residents and residents were actively involved; preprogrammed music made residents reminisce; the robot added value for one-to-one situations (provoke interactions, emotions and stimulate clients); the robot stimulated residents to move, provides reactions from residents |
| Hung et al., 2022, Canada [11] | - Journal article - Qualitative study; semi-structured interviews | - Two long-term care sites - 30 long-term care frontline interdisciplinary healthcare providers, operational leaders, residents and family members, ethics experts in dementia care | - Social robot PARO - PARO elicits emotive responses and serves as a social companion | - Healthcare providers worried that the robot could be used as a weapon in situations that involved behavioural events - The cognitive and physical disabilities of residents made safety risk a priority concern for healthcare providers - Concerns about the freedom to decline robot use by residents with dementia - With current healthcare providers shortage: perceived issues with extra work required for healthcare providers (charge, wipe, disinfect, maintain, and repair the robots, teach new healthcare providers and families about the robot) - Time required to provide education and ongoing support - Robots would not be used without support or training to use it - Nervous about the cost of the robot - Worried about robots replacing human care and visits | To prepare and provide:   - Risk assessments and risk management to avoid resident conflict/violence to prevent injury among residents - Practical recommendations on how to train and prepare healthcare providers, residents, and families effectively - Appropriate resources to balance workload   Positive perception from healthcare providers:   - Healthcare providers agreed that robots could offer support for social connection in long-term care |
| Jonas, 2022, Canada [94] | - Online news article - Pilot study, method not specified, collecting feedback from residents about interacting with the robot | - Nursing home - Residents and healthcare providers, number not specified | - Humanoid robot Grace | N.A. | - The robot could help with healthcare providers’ workload in interacting with residents - The robot could help to improve the mental well-being of residents by interacting with them |
| Kolstad et al., 2020, Japan [86] | - Journal article - Qualitative study | - 3 nursing homes - Facilities' managers and nursing healthcare providers | - Socially Assistive Robots (Social robot PARO, Humanoid robot Pepper) - Communication robots Pepper is used for recreation, entertaining patients through games and karaoke | - Patients found Pepper loud and annoying - Patients that were not cognitively capable might find PARO less interesting - Required human workers and attention as the robots were not fully independent | - All robots worked with the mental wellbeing of the patients - Patients had an emotional relationship with PARO |
| Kriegel et al., 2019, Austria [95] | - Book chapter - Literature search; online questionnaires; expert interviews | - Different care settings including LTC homes - Expert interviews: 6 (1 nursing home director, 2 nurses, 3 graduate social workers for elderly work, 1 kitchen manager, 1 laundry assistant); online survey: 46 nursing home directors | - Socially assistive robots - To support in-patient care for the elderly through social interaction and human users (such as employees, residents; Function-related support processes (e.g., transport of food, laundry, care supplies); resident-related processes (e.g., communication, entertainment, therapy support) | - Lack of support services for technologies in the context of in-patient care for the elderly on: - integration and maintenance of existing and required software and information technologies - interface management between the different software programs - Other challenges: threats of data abuse, healthcare providers feeling being monitored by social assistive robots and artificial intelligence | - Important to consider the technical, legal, economic, psychological, social, and ethical dimensions of the technologies - Provision of the appropriate interfaces, standards, and necessary infrastructures for embedding the social assistive robots in the complex supply system - The embedding of the social assistive robot technologies into the existing service processes - Provision of the training and involvement of the employees and residents involved - Respective acceptance on the part of employees, residents, and relatives towards social assistive robots - Availability of dedicated social assistive robots’ services providers to enable and ensure hybrid services in nursing homes - Solutions and strategies adapted to the respective structural and process-related circumstances and preferences |
| Lehmann et al., 2021, Switzerland [81] | - Conference proceeding - Semi-structured telephone interviews; a self-compiled questionnaire | - A nursing home - 5 caregivers in a nursing home (the ward manager and the safety officer) | - Social robot PARO - Assist in the care of older adults - Provide companionship | - Shape of the robot: - caregivers found it difficult to imagine how the residents would react to the headless shape of the robot - Difficulties were seen in the aspect of hygiene - Healthcare providers perceived that: - residents might have less human contact - residents might be afraid of the robots - residents might be deceived ethically | N.A. |
| Li et al., 2022, Canada [72] | - Journal article - Surveys | - Long-term care home - 2 Robots and 81 participants into 10 sessions (5 resident and 5 healthcare providers) with approximately 8 people in each session | - Humanoid robots Pepper and NAO - Entertainment | - Concerns regarding training on how to properly use the robot and the added workload related to operating the robot - Concerns about the robot not being able to operate independently without supervision and being used only as an additional helper | - Both the healthcare providers and the resident groups had positive perceptions and attitudes towards the robots for the dance activity: - Dance therapy with robot made possessive effect on residents - Residents thought it was easy for them to follow the robots during the dance sessions - Suggestions from staff related to the future deployment logistics of such robots |
| McGinn et al., 2019, United States [49] | - Journal article - Qualitative and quantitative mixed study; thematic analysis | - Long-term care home - 2 female senior nursing healthcare providers | - Social robots, Stevie - Communication | - Noise of robot - Nonsterile surface material | N.A. |
| Melkas et al., 2019, Finland [71] | - Journal article - Qualitative study | - 2 care homes and 1 geriatric rehabilitation hospital - 35 care workers (nurses, assistant nurses, physiotherapists, and occupational therapists) | - Humanoid robot NAO - Rehabilitation and recreational assistance, like exercise, music, games, dances, storytelling | - Time-consuming workflow integration, with personal resources required - Internet connection and storage and charging space required - Robot with a small size and low voice, which was not friendly to clients with eyesight and hearing impairment - Threat from robots to the sense of control at work - Conflicts spurred between healthcare providers working and those playing with robots | - Ample time for orientation and training to all healthcare providers |
| Mitzner et al., 2018, United States [75] | - Journal article - Quantitative and qualitative study | - Residential nursing facilities - 14 healthcare providers | - Service robot PR2 - Assisting with caregiving tasks (light housework, IV use, infusion pump devices, dispensing medications, transfer) | - Afraid of healthcare providers’ job replaced by robots - Afraid of possibility of making mistakes by the robots, especially some direct tasks for patients, like bathing and dressing | N.A. |
| Obayashi et al., 2020, Japan [74] | - Journal article - Quantitative and qualitative study | - Residential care home - 25 older people with 15 healthcare providers members (13 nurses and 2 care workers) | - Social robot Sota - Monitoring sleeping of resident by connecting to sheet-shaped body vibrometer (SBV) | - Flashing eye of robot at night might frighten residents - Perceived robots as an interruption as robots might wake up residents at night - Residents might lose interests due to robots’ failure to build effective communication | N.A. |
| Papadopoulos et al., 2022, United Kingdom [70] | - Journal article - Qualitative study; semi-structured interviews | - Care homes - 23 care home workers | - Humanoid robot Pepper - Communication | - Robots not having all human traits and communication - Robots restricted to verbal style instead of other non-verbal communication - Older people were slow to adapt to new technology and they might develop negative emotions such as fear - Concerns related to the possibility of jobs being replaced by robots - Concerns over privacy of residents - Care providers were not equipped with enough knowledge related to robot, like scope and function, operation, and maintenance | - More systematic education and training in detail should be standardized in long-term care homes before robots are introduced - Familiarity with robots will maximize the benefits it can provide in long-term care settings and reduce care workload |
| Pedell et al., 2022, Australia [96] | - Book chapter - Qualitative study; participatory design; case study | - Long-term care home, men's shed - 60 residents - Healthcare providers (number not specified) | - Social Robot PARO - Communication | - Unfamiliarity with robot | - Invites residents to make clothes for the robots by knitting and crocheting (to help remove hesitation and fear when facing the robot by building connection) - Enable robot’s responses to injuries (to stimulate the emotional response of residents and help enhance connection between them) |
| Pfadenhauer and Dukat, 2015, Germany [69] | - Journal article - Video-assisted Ethnographic study | - Nursing home - video footage, photos, informal conversations and team discussions, and observation (number of staff participated not specified) | - Social robot PARO - Communication and observation instrument | - Unable to move the robot into the room independently - Fear of the robot could replace human manpower | - Equip staff with carrying techniques of the robots |
| Robinson et al., 2016, New Zealand [77] | - Journal article - Part of a randomized-controlled trial; this study only includes a session with observation and interview | - Aged care facility - 16 residents and 21 healthcare providers | - Social robot PARO - Companion for loneliness, relaxation, and socialization. | - Residents not interested in the robot because to them it was like a toy and not appealing to all residents | - The robot should be altered to be smaller, lighter, with a different colour, and be able to interact more and purr |
| Shin et al., 2015, Switzerland [88] | - Conference proceeding - Interviews and surveys | - Retirement homes - 23 residents and 8 healthcare providers | - Robot Smartwalker - Evaluates the appropriateness and usefulness of the walker and its gesture-based interface for the elder | - Improvements needed for the walker | - Improvements suggested: - Uphill/downhill support - A parking brake for safety - An ergonomically designed tiltable handgrip - Obstacle and stair recognition for warning, and more audible warnings |
| Tanioka et al., 2019, Japan [97] | - Journal article - Qualitative study (participatory action research method) | - Long-term care homes - Number of healthcare providers interviewed not mentioned | - Humanoid robot Pepper - Motivate for older adults to perform exercise and activities | - Required support from healthcare providers for patients according to patients’ level of cognitive function | N.A. |
| Moyle et al., 2018, Australia [80] | - Journal article - Descriptive qualitative study, cluster-randomized-controlled trial with another toy without AI | - Long-term care facility - 20 care healthcare providers | - Social robot PARO - Interaction | - Worried about the cost - Concerns regarding healthcare providers’ training - Concerns over ethical issues, for example, residents’ feeling of being treated as a child | N.A. |
| Louie et al., 2014, Canada [78] | - Conference proceeding - Qualitative study | - Long-term care home - 43 individuals (health care healthcare providers and families) | - Social robot Tangy - Plan, schedule, and facilitate social and cognitive interventions: group recreational and one-on-one telepresence activity | - Mechanical appearance and synthesized voice - Lack of experience with modern technology, for example, some residents was fear of the robot because of their unfamiliarity with technology - Language barriers as half of the residents were non-English speakers | - Dress robots with clothes - Add translation functions - Incorporate reminding and prompting functions on game schedules, meals, and missed calls |
| Louie, 2017, Canada [90] | - PhD dissertation - Mixed study design with qualitative and quantitative study | - Long-term care home - Older adults and formal caregivers | - Social robot Tangy - Support multi-user activities, learn from caregivers, improve compliance by personalizing robot behaviours | - The robot was relatively slow on picking up healthcare providers’ instructions | Healthcare providers positive perception:   - The robot was easy to use - Low workload when using the robot - Different possible uses of the robot - Using the robot was enjoyable - The robot can be customized to residents’ diverse needs |
| Yuan et al., 2022, Australia [84] | - Conference proceeding - Semi-structured interviews | - 6 residential aged care organizations - 11 healthcare providers | - Social robots PARO, Humanoid robot NAO - Increase communication and evoke caring behaviours | - If the healthcare providers did not take the robots seriously, for example, treating them as toys, the probability of the older adults’ unacceptance towards the robots would increase | - Provision of appropriate training and to engage healthcare providers to run programs with robots - Provision of training manual that was easy to follow by healthcare providers with low level of digital literacy - Healthcare providers have sufficient knowledge about the robots - Engagement of people interested in technology to take on the role as key technology champions - Respected residents' perceptions of robopets while using them (for example, treating robot pets as real as how the residents though; using Nao to meet emotional needs of residents who could not speak English and program songs in their language) - Healthcare providers are active in the initiation of human-robot interaction and to guide the care that the robot can perform: - Created meaningful activities with the robot (NAO) for residents (e.g., doing a concert with the robot) - Involved NAO in residents' daily routines - Adapted the use of robots to individual needs (e.g., embed residents' past relationships with animals into their personalized care plans) - Match the characters of robots to the preferences of residents to enhance the robots' effective use - Use robopets in one-to-one activities to avoid residents' conflicts of the ownership of robopets in group activities - Healthcare providers recognized the potential benefits and values of the robots: - robotic pets had significant social and emotional benefits for residents living with dementia - reduce care healthcare providers job stress - expand healthcare providers’ opportunities for providing social care - increase healthcare providers’ satisfaction |
